# Hijacking of the AP-1 Signaling Pathway during Development of ATL

**DOI:** 10.3389/fmicb.2017.02686

**Published:** 2018-01-15

**Authors:** Hélène Gazon, Benoit Barbeau, Jean-Michel Mesnard, Jean-Marie Peloponese

**Affiliations:** ^1^Belgium Molecular and Cellular Epigenetics, Interdisciplinary Cluster for Applied Genoproteomics, University of Liège, Liège, Belgium; ^2^Département des Sciences Biologiques and Centre de Recherche BioMed, Université du Québec à Montréal, Montréal, QC, Canada; ^3^Institut de Recherche en Infectiologie de Montpellier, Centre National de la Recherche Scientifique, Université de Montpellier, Montpellier, France

**Keywords:** AP-1, HTLV-1, antisense transcription, leukemia, HBZ, JunD

## Abstract

Human T-cell leukemia virus type 1 (HTLV-1) is the causative agent of a fatal malignancy known as adult T-cell leukemia (ATL). One way to address the pathology of the disease lies on conducting research with a molecular approach. In addition to the analysis of ATL-relevant signaling pathways, understanding the regulation of important and relevant transcription factors allows researchers to reach this fundamental objective. HTLV-1 encodes for two oncoproteins, Tax and HTLV-1 basic leucine-zipper factor, which play significant roles in the cellular transformation and the activation of the host’s immune responses. Activating protein-1 (AP-1) transcription factor has been linked to cancer and neoplastic transformation ever since the first representative members of the Jun and Fos gene family were cloned and shown to be cellular homologs of viral oncogenes. AP-1 is a dimeric transcription factor composed of proteins belonging to the Jun (c-Jun, JunB, and JunD), Fos (c-Fos, FosB, Fra1, and Fra2), and activating transcription factor protein families. Activation of AP-1 transcription factor family by different stimuli, such as inflammatory cytokines, stress inducers, or pathogens, results in innate and adaptive immunity. AP-1 is also involved in various cellular events including differentiation, proliferation, survival, and apoptosis. Deregulated expression of AP-1 transcription factors is implicated in various lymphomas such as classical Hodgkin lymphomas, anaplastic large cell lymphomas, diffuse large B-cell lymphomas, and adult T-cell leukemia. Here, we review the current thinking behind deregulation of the AP-1 pathway and its contribution to HTLV-induced cellular transformation.

## Introduction

The human T-cell leukemia virus type 1 (HTLV-1) was the first pathogenic retrovirus identified in human (Matsuoka and Jeang, 2010). It is estimated that 10–15 million individuals are infected with HTLV-1 around the world, with endemic areas in the Caribbean, southern Japan, Central and South America, Iran, Melanesia, and sub-Saharan Africa (Sonoda et al., 2011; Gessain and Cassar, 2012). While the vast majority of HTLV-1-infected individuals remain clinically asymptomatic, around 5% of them will develop a highly aggressive T-cell malignancy, termed adult T-cell leukemia/lymphoma (ATL) (Matsuoka and Jeang, 2007; Kogure and Kataoka, 2017). ATL presents four distinct clinical stages ranging from smoldering to acute leukemia. It generally occurs in individuals infected around the time of birth eventually and it develops only after prolonged incubation periods ranging from 20 to 60 years (Matsuoka et al., 1997). Although several studies have reported that the proviral DNA load is a critical factor for promoting disease progression in infected individuals (Olindo et al., 2005; Iwanaga et al., 2010; Yoshida, 2010), 30 years after its characterization in T-lymphocytes from leukemic patients, it is still not fully understood how HTLV-1 transforms human CD4+ T cells in a stepwise fashion. The current view is that pleiotropic functions of the HTLV-1 viral transcriptional transactivator Tax (Peloponese et al., 2007; Journo et al., 2009), such as deregulation of the signaling pathways AP-1 pathway (Fujii et al., 2000) and NF-kB (Rosin et al., 1998; Peloponese et al., 2006; Chan and Greene, 2012), and inactivation of tumor suppressors (Tabakin-Fix et al., 2006) are promitotic events, which drive CD4+ T-cell proliferation during the preleukemic stage (Matsuoka and Jeang, 2007). Paradoxically, fresh ATL cells lack Tax expression, due to genetic and epigenetic modifications in the HTLV-1 provirus (Tamiya et al., 1996; Kataoka et al., 2015). In contrast, HTLV-1 basic leucine-zipper factor (HBZ) mRNA which is encoded by the complementary strand of the HTLV-1 genome is expressed in all ATL cells (Mesnard et al., 2006; Matsuoka and Green, 2009). Recent studies have provided striking evidence for the important role played by of HBZ and the AP-1 pathway in HTLV-1 pathogenesis. In this review, we will limit our focus to the role of AP-1 activation by Tax and HBZ and discuss, in a non-exhaustive manner, how this activation relates to oncogenesis and inflammation.

## The AP-1 Pathway, A Key Regulator of Cellular Transformation

**FIGURE 1:**
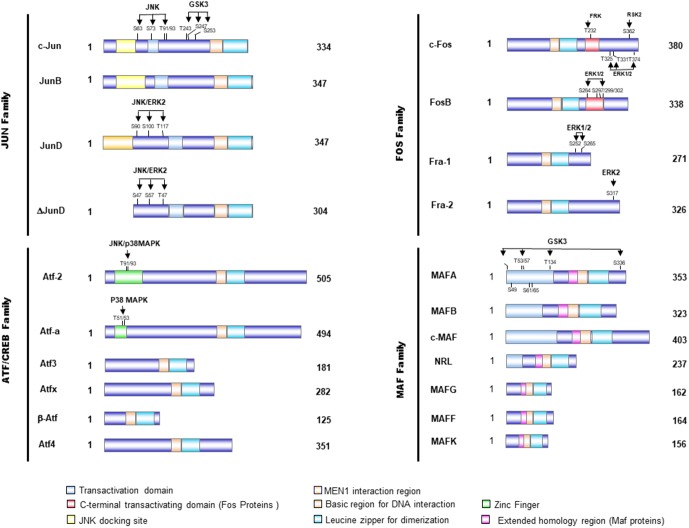
Schematical presentation of the structure of AP-1 proteins. Activator protein 1 (AP-1) proteins include the JUN, FOS, activating transcription factor (ATF), and musculoaponeurotic fibrosarcoma (MAF) protein families, which can form homodimers and heterodimers through their leucine-zipper domains. The AP-1 proteins exhibit several domains, including the bZIP domain (leucine zipper plus basic domain), transactivation domains, and docking sites for several kinases, such as JNK or ERK. These kinases modulate the activity of those transcription factors by phosphorylation of serine and threonine residues.

**FIGURE 2:**
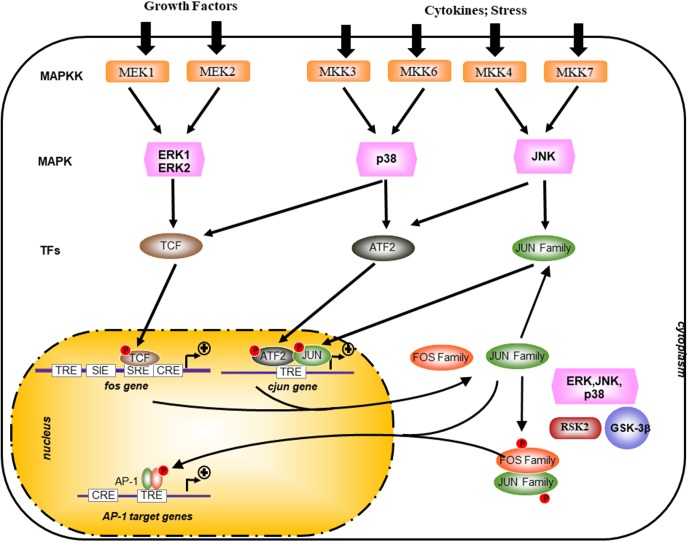
Transcriptional and post-translational activation of AP-1. AP-1 activity is stimulated by external stimuli like growth factors or inflammatory cytokines and a complex network of kinase such as mitogen-activated protein kinases (MAPKs) of the extracellular-signal regulated kinase (ERK), p38, and JUN amino-terminal kinase (JNK) families. Posttranslational phosphorylation by various kinases regulates AP-1 activity, which includes its transactivating potential, DNA-binding capacity, and the stability of AP-1 components. GSK-3β, glycogen synthase kinase-3β; MAPKK, MAPK kinase; RSK2, ribosomal S6 kinase 2; TCF, ternary complex factor; SRE, serum-response element; TRE, TPA-responsive element; CRE, cAMP-response element; SIE, Sis-inducible element.

Activating protein-1 (AP-1) transcription factor has been linked to cancer and neoplastic transformation since the first cloning of *jun* and *fos* proto-oncogenes were cloned following their identification as cellular homologs of avian sarcoma virus 17 (ASV 17)-encoded oncogenes *vjun* and *vfos* 30 years ago (Curran and Franza, 1988). AP-1 is composed of 18 dimeric complexes which included members of four families of DNA-binding proteins: Jun family (c-Jun, JunB, v-Jun, JunD), Fos family (c-Fos, FosB, Fra-1, and Fra-2,) ATF/cyclic AMP-responsive element-binding (CREB) (activating transcription factor: ATF1–4, ATF-6, b-ATF, ATFx), and Maf family (musculoaponeurotic fibrosarcoma c-Maf, MafA, MafB, MafG/F/K, and Nrl) (Eferl and Wagner, 2003; Milde-Langosch, 2005; Hernandez et al., 2008; **Figure 1**). Transcriptional activity of AP-1 is regulated by a wide array of cellular stimuli including growth factors, bacterial and viral infection, cytokines, UV radiation, and cellular stress (Eferl and Wagner, 2003; Milde-Langosch, 2005; Hernandez et al., 2008; **Figure 1**). These transcription factors have critical functions in wide variety of cellular processes, including inflammation, proliferation, differentiation, and apoptosis (Eferl and Wagner, 2003; Milde-Langosch, 2005; Hernandez et al., 2008). The activity of the different AP-1 dimer also depends on the cell type and its differentiation state. In response to external stimuli, MAPK activity increases and regulates both the abundance and transactivating capacities of Jun, Fos, and ATF (**Figure 2**). MAPKs are a serine/threonine kinase superfamily that comprises extracellular signal-regulated kinases (ERK), c-Jun NH2-terminal kinases (JNK), p38, and c-Fos-regulating kinases (FRK) (Cavigelli et al., 1995; Karin, 1995; Srivastava et al., 1999). The regulation of AP-1 is complex and occurs at multiple levels, ranging from dimer composition, to transcriptional and post-translational events, and to specific interactions between AP-1 proteins and other transcription cofactors (**Figure 2**).

### AP-1 Transcriptional Regulation

Activating protein-1 activity is modulated through its dimer composition which is determined by the differential expression of Jun, Fos, ATF, and Maf families (**Figure 1**) and through the sequence of the AP-1 DNA-binding sites (**Table 1**). The abundance of the subunits can be controlled either via the regulation of the synthesis and stability of respective mRNAs or via the regulation of protein stability (for example, stimulus-dependent degradation via the ubiquitin pathway) (Musti et al., 1996). Most of the genes that encode AP-1 subunits behave as “immediate-early” genes. Indeed, they are rapidly but transiently transcribed in response to extracellular stimuli, such as growth factor (Ryder and Nathans, 1988; Karin et al., 1997) and cellular stress (Angel and Karin, 1991; Zhou et al., 2007; **Figure 2**). Among these, the transcriptional regulation of *c-jun* and *c-fos* is well studied and characterized (Abate and Curran, 1990). The transcription of *c-fos* is induced in response to a diverse spectrum of extracellular stimuli and its promoter is composed of several transcription factor-binding sites, such as a cAMP-response element (CRE), which can drive transcriptional activation in response to elevation of intracellular Ca^2+^ or cAMP concentrations under stimulation from neurotransmitters and polypeptide hormones (**Figure 2**; Lucibello et al., 1993; Cavigelli et al., 1995; Tulchinsky, 2000). It also contains a serum-response element (SRE), which can drive transcription in response to growth factors, cytokines, UV irradiation, and other stimuli. SRE is recognized by a dimer composed of serum-response factor (SRF) and Elk-1, the major component of ternary complex factor (TCF) in human cells (Lucibello et al., 1993; Cavigelli et al., 1995; Tulchinsky, 2000; **Figure 2**). The third major element of c-Fos promoter is the v-Sis-inducible element (SIE) (Wagner et al., 1990). SIE is mostly recognized by homodimers and heterodimers of STAT1 and STAT3, two members of the signal transducers and activators of transcription (STAT) family (Sadowski et al., 1993). Tyrosine phosphorylation of these factors in the cytoplasm is mediated by janus kinase/tyrosine kinase (JAK/TYKs) and drives their dimerization. The dimerized factor can then translocates to the nucleus, binds the SIE, and participates in promoter activation. Finally, the c-Fos promoter also contains a 12-*O*-tetradecanoyl-phorbol-13-acetate-response element (TRE) (**Figure 2**; Lucibello et al., 1993; Cavigelli et al., 1995; Tulchinsky, 2000).

**Table 1:** The different AP-1-binding sites.

	AP-1-binding sequence
TRE	TGACTCA
MAREI	TGCTGACTCAGCA
CRE	TGACGTCA
MARE II	TGCTGACGTCAGCA
ARE	a/gtGACnnnGC

*The main DNA response element is the TPA-responsive element (TRE), but different dimers preferentially bind to elements such as the cAMP-response element (CRE), the MAF-recognition elements (MAREs), and the antioxidant-response elements (AREs)*.

The *c-jun* promoter is simpler, being mostly induced through the TRE element that preferentially binds c-Jun/ATF2 heterodimers (**Figure 2**; Van Dam et al., 1993). Despite its inducible expression, most cell types prior to their stimulation contain basal levels of c-Jun protein. Like the *c-fos* SRE, the *c-jun* TRE is constitutively occupied *in vivo* (Van Dam et al., 1993). Thus, the expression of more than one AP-1 component is under positive and negative AP-1 (auto-)regulation. For example, *c-jun* and *atf3* promoters can be activated by c-Jun/ATF2 and/or ATF2/ATF2 via TRE-binding sites, whereas the *atf3* promoter is inhibited by ATF3 (Angel et al., 1988; Hai and Curran, 1991; Van Dam et al., 1993). The *c-jun* promoter can be inhibited by JunB, c-Jun itself, and c-Fos. This feedback control allows fine-tuned regulation of AP-1 heterodimer activity over longer periods of time (Chiu et al., 1988).

### Post-transcriptional Regulation of AP-1 Transcription Factors

Phosphorylation of AP-1 components modulates the dimers transcriptional activities (Karin and Hunter, 1995). Serum and growth factors stimulation induces AP-1 by activating the ERK which then directly phosphorylate c-Jun, Fra-1, and Fra-2 (**Figure 2**). While phosphorylation of c-Jun by ERK on one serine located next to the C-terminal DNA-binding domain inhibits c-Jun DNA-binding activity, phosphorylation of Fra-1 and Fra-2 enhances their DNA binding in conjunction with c-Jun (Woodgett et al., 1993; Punga et al., 2006).

The induction of AP-1 by pro-inflammatory cytokines and genotoxic stress is mostly mediated by the JNK and p38MAPK pathways (Chang and Karin, 2001; **Figure 2**). Once activated, JNKs translocate to the nucleus, where they phosphorylate c-Jun on Ser 63/73 and Thr 91/93 and thereby potentiates its ability to activate transcription upon homodimerization or a heterodimerization with c-Fos (Woodgett et al., 1993; Deng and Karin, 1994; Punga et al., 2006). The molecular mechanisms underlying the capacity of JNK to control c-Jun activity involve the modulation of interactions with histone deacetylase complexes, sub-nuclear localization of AP-1 proteins, and related factors required for c-Jun-dependent activity. JNKs also phosphorylate ATF2 on Thr69/71 and potentiate its activity after heterodimerization with c-Jun, leading to its binding to divergent AP-1 sites in the *c-jun* promoter (Van Dam et al., 1993; Shaulian and Karin, 2002). Transactivation by ATF2 is also potentiated by binding of retinoblastoma (Rb) or E1A, to the DNA-bound ATF2 dimer (Lopez-Bergami et al., 2010). Both E1A and Rb act in concert with phosphorylation of ATF2. Although E1A induces c-jun transcription (Van Dam et al., 1993), it concomitantly represses AP-1 activity through competition for CREB-binding protein (CBP) in a similar manner to the competitive effect of E1A binding on p300 (Offringa et al., 1990; Arany et al., 1994).

The contribution of p38 to AP-1 induction can be mediated by the direct phosphorylation and activation of ATF2 and TCFs (**Figure 2**; Mendelson et al., 1996; Whitmarsh and Davis, 1996). The PI3K/AKT pathway is activated in response to cytokine receptors and T-cell receptor activation in normal T cells. Akt is a serine/threonine protein kinase activated by PI3K through phosphorylation of Ser473, which acts as a regulator of cell survival and proliferation (Warfel and Kraft, 2015). In addition, glycogen synthase kinase-3 (GSK3), an effector kinase of the PI3K pathway, has the capacity to negatively regulate AP-1 transcriptional activity (Koul et al., 2007; Warfel and Kraft, 2015). GSK3 is a ubiquitously expressed serine/threonine kinase normally active in unstimulated cells. Upon stimulation by growth factors, GSK3 is phosphorylated at Ser9 and Ser21 (for GSK3β and GSK3a, respectively) by Akt and other kinases of the AGC family (protein kinase A, protein kinase G, protein kinase C) thus leading to an important decrease of its activity (Koul et al., 2007; Venkatesan et al., 2010). GSK3 activity is controlled mainly through the PI3K/AKT pathway upon AKT phosphorylation on Ser473 (Koul et al., 2007). This complex network of signaling pathways reveals that a particular stimulus can evoke a specific “spectrum” of AP-1 activity and thereby activate and/or repress distinct subset of AP-1-targetted genes.

### The Janus (Dual) Role of AP-1 in Cancer Development

A large amount of studies have shown that AP-1 components play an important role in oncogenesis. *c-jun* and *c-fos* were first identified as retrovirus-activated genes with oncogenic potential in avian and mammalian cells (Abate and Curran, 1990; Verma et al., 1990). Chronic exposure to carcinogens can promote tumorigenesis through the activation of a wide array of signaling pathways, ranging from inflammatory to pro-proliferative and survival pathways. Furthermore, environmental or dietary carcinogens have been shown to induce increased AP1 activity (Abate and Curran, 1990; Verma et al., 1990).

Many human cancers exhibit overexpression of Jun family members (Neyns et al., 1996; Langer et al., 2006; Kharman-Biz et al., 2013). Consistent with the idea that c-Jun can promote tumorigenicity, overexpression of this transcription factor is observed in some of the more aggressive CD30-positive lymphomas (Drakos et al., 2007; Mao et al., 2007). In breast cancer, alteration of RB, VEGF, and EGFR pathways has been shown to induce c-Jun overexpression (Kharman-Biz et al., 2013). Interestingly, increased c-Fos expression is associated with poor clinical outcome in osteosarcoma and endometrial carcinoma, while loss of c-Fos expression is associated with tumor progression and adverse outcome in ovarian carcinoma and gastric carcinoma (Tulchinsky, 2000). On the other side, Fra-1 overexpression is associated with the development of thyroid, breast, lung, brain, nasopharyngeal, esophageal, endometrial, prostate, and colon carcinomas, along with glioblastomas and mesotheliomas (Tulchinsky, 2000; Young and Colburn, 2006). The studies are strongly suggesting that the role of Fos family in tumors development depends on the tissue of origin.

Several studies have shown that AP-1 activity is crucial for tumorigenesis, as its inhibition by dominant-negative c-Jun mutants or AP-1 decoys strongly inhibits the growth of various tumor cell lines both *in vitro* and *in vivo* (Angel and Karin, 1991; Kajanne et al., 2009; Eckert et al., 2013; Kharman-Biz et al., 2013). These studies have also led to the identification of AP1 target genes involved in carcinogenesis (Eferl and Wagner, 2003; Lopez-Bergami et al., 2010; Nakayama et al., 2012). In addition, chronic exposure to environmental and dietary carcinogens such as cigarette smoke or nicotine or ethanol, activates AP1 activity in mouse brain or epithelial cell lines or neuroblastoma cells (Fried et al., 2001; Jochum et al., 2001; Manna et al., 2006). Interestingly, increase in AP1 activity has been also reported in drug-resistant cancer, suggesting that some chemotherapeutic agents can elicit AP1 activation and favor tumor cell survival by making them refractory to long-term treatments (Malorni et al., 2016; Fan et al., 2017; Liou et al., 2017).

Overexpression of Jun and Fos proteins can also suppress tumor formation (Eferl and Wagner, 2003; Hess et al., 2004; Shaulian, 2010), thus revealing the double-edged activity of AP-1 transcription factors. These dual properties depend on the genetic background of the tumor, its differentiation state, and tumor stage (Eferl and Wagner, 2003; Hess et al., 2004; Shaulian, 2010). Several studies have shown that increased AP-1 activity can lead to apoptosis in human tumor cells but it can also antagonize apoptosis in specific cell types, such as liver tumors (Eferl and Wagner, 2003; Hess et al., 2004; Shaulian, 2010). This dual effect of AP-1 on apoptosis can be further exemplified. Indeed, increased c-Jun activity promotes apoptosis in neuronal cells *in vitro* (Ham et al., 2000). When the activation of c-Jun is impaired, either in Jnk3-null or JunAA mice, which express a c-Jun insensitive to JNK-mediated activation, neurons are protected from apoptosis (Behrens et al., 1999). In contrast, c-Jun is required for the survival of fetal hepatocytes, which undergo apoptosis in c-Jun-deficient mouse embryos (Eferl et al., 1999; Hasselblatt et al., 2007). The cell-specific consequence of AP-1 activity over apoptosis is likely due to its differential regulation of pro-apoptotic and anti-apoptotic target genes. In neurons, c-Jun regulates the expression of Bim, a pro-apoptotic Bcl-2 family member that is crucial for neuronal apoptosis. While in T cells, c-Jun regulates the expression of Fas ligand (FasL), which upon binding to the Fas receptor triggers apoptosis (Eferl and Wagner, 2003; Hess et al., 2004; Shaulian, 2010). Bcl-2 family members are also part of the list of anti-apoptotic targets that are regulated by AP-1 (Kirkin et al., 2004). In T cells, Jun members exert a protective signal through the induction of Bcl-3, while in myeloid cells, inactivation of JunB leads to reduced apoptosis with increased expression of the anti-apoptotic Bcl-2 gene (Kyriakis, 1999; Srivastava et al., 1999; Kirkin et al., 2004) have shown that hepatocytes deficient for c-Jun or JunD are highly sensitive to tumor necrosis factor-α-induced apoptosis, thus suggesting that c-Jun and JunD might regulate genes that protect cells from TNF-α-induced-cell death. Those studies have demonstrated that depending on the type of extracellular stimuli and on the cellular context, activation of AP-1 can have a different outcome on the cell fate. Therefore, the role and function of AP-1 in cancer development should be examined within the context of a complex network of simultaneously triggered signaling pathways.

## Regulation of AP-1 During HTLV-1 Infection

Human T-cell leukemia virus type 1 can infect a variety of cell types *in vivo*, including T cells, B cells, and macrophages (Jones et al., 2008; Pique and Jones, 2012; Gross and Thoma-Kress, 2016; Rizkallah et al., 2017). The HTLV-1 provirus is detected mainly in CD4+ T cells and to lesser extent in CD8+ T cells (Richardson et al., 1990; Iwahashi et al., 1991; Melamed et al., 2015). This asymmetry in detection may be caused by recruitment of CD4+ T cells and induction of their proliferation following HTLV-1 infection in contrast to a delayed cell death in CD8+ T cells (Sibon et al., 2006; Alais et al., 2015). A subset of HTLV-1-infected individuals will develop ATL after an extended period of time (Matsuoka and Jeang, 2007; Kannian and Green, 2011). Infected cells, however, must initiate proliferation and evade apoptosis as a prelude to immortalization and transformation. Virally encoded oncogenic proteins are known to dysregulate various cellular pathways or processes through the regulation of the activity of target proteins. HTLV-1 transforms T cells via its transactivator Tax, which interferes with pathways regulating cell growth control through activation of various cellular transcription factors (NF-κB, E2F, and AP-1) (Fujii et al., 2000; Peloponese et al., 2006; Journo et al., 2009) and inactivation of p53 (Tabakin-Fix et al., 2006). Since AP-1 has been implicated in transformation of T cells, it has been hypothesized that inappropriate activation of AP-1 could contribute to the dysregulated phenotype of HTLV-1-infected cells or to the development of ATL. Indeed, ATL leukemic cells exhibit increased levels of mRNAs encoding JunD and Fra-2 (Nakayama et al., 2008; Terol et al., 2017) and high levels of AP-1-binding activity (Fujii et al., 2000; Iwai et al., 2001), with in addition, AP-1, more specifically c-Jun/c-Fos heterocomplex, induces HTLV-1 promoter activity and thus participates in HTLV-1 basal transcription (Jeang et al., 1991).

### Activation of the AP-1 Pathway by Tax during Acute HTLV-1 Infection

The phosphoprotein Tax is encoded within the pX region of HTLV-1 genome and is a viral regulatory protein. This protein mainly localizes to the nucleus and is a well-known trans-activator of the HTLV-1 long terminal repeat (LTR) operating through three 21-bp repeats called Tax-responsive elements (TxREs) (Meertens et al., 2004; Grassmann et al., 2005). Its pleiotropic properties confer a pivotal role for Tax toward viral pathogenicity and immortalization/transformation of infected cells, causing the onset of HTLV-1-associated diseases (Giam and Jeang, 2007; Peloponese et al., 2007). Indeed, the protein is sufficient for immortalizing primary human T cells and rodent fibroblasts as well as inducing tumors in nude mice inoculated with Tax-transformed cells (Grassmann et al., 2005). Furthermore, Tax interferes with important functions, leading to cell cycle dysregulation and promoting *in vivo* clonal expansion (Giam and Jeang, 2007; Peloponese et al., 2007). Moreover, Tax can alter the expression of cellular proteins involved in cell growth and proliferation such as cytokines (Grassmann et al., 2005; Matsuoka and Jeang, 2010). Importantly, this trans-acting factor also acts as a transcriptional regulator of gene expression by recruiting or modifying the activity of cellular transcription factors (such as CREB protein, SRF, NF-κB, and notably AP-1) through direct or indirect interactions (Grassmann et al., 2005; Matsuoka and Jeang, 2010).

**FIGURE 3:**
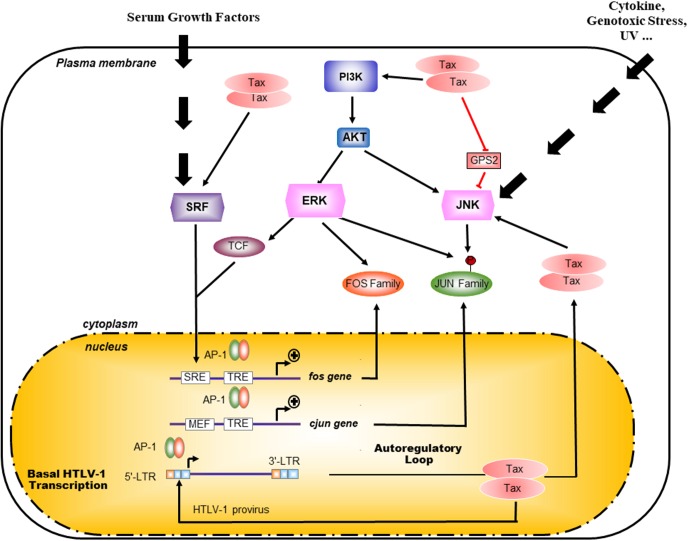
Mechanisms of Tax activation of AP-1 pathway. Pathways upstream of AP-1 are normally activated in response to external stimuli whereas presence of Tax overrides this requirement. Indeed Tax is able to activate the Akt/PI3K pathway as well as the SRF pathways thus activating at the transcription of c-Fos, Fra-1, c-Jun, JunB, and JunD genes. By interacting with the JNK inhibitor G-protein pathway suppressor 2 (GPS2), Tax also participate to the exhibited highly activity of AP-1 in HTLV-1 infected cells through a constitutive activation of JNK (Jin et al., 1997). Interestingly, AP-1 proteins such as c-Jun and c-Fos activate the transcription through the 21 bp repeat in HTLV-1 LTR.

Tax activates the transcription of cellular genes by activating AP-1 DNA binding to promoter elements in T cells (Fujii et al., 2000; Iwai et al., 2001; **Figure 3**). Thus, activation of AP-1 by Tax is thought to contribute to the deregulated phenotypes and leukemogenesis of T cells infected with HTLV-I. Among these factors, c-Fos, Fra-1, c-Jun, JunB, and JunD genes have been shown to be activated by Tax at the transcriptional level (Fujii et al., 2000; Iwai et al., 2001; **Figure 3**). Interestingly, exogenous expression of Tax in Jurkat cells induced AP-1-dependent transcription of a reporter gene more efficiently than any combinations of AP-1 proteins (Fujii et al., 2000; Iwai et al., 2001). Thus, the well-known induction of expression of multiple Fos and Jun family members by Tax is known to be essential, but may not be sufficient, for the transcriptional activation of AP-1 sites mediated by Tax (Fujii et al., 2000; Iwai et al., 2001). Since DNA-binding activity and transcriptional activation of AP-1 are regulated at a post-translational level, Tax might be involved in this regulation (Fujii et al., 2000; Iwai et al., 2001). Tax contributes to the high activity of AP-1 in HTLV-1-infected cells through different mechanisms with intricate ramifications in cascade signaling (**Figure 3**). A constitutive activation of JNK had initially been reported in HTLV-1-infected cells, such as Tax-expressing MT-2 cells and Tax-ATL primary cells. Furthermore, this activity seemed to depend on the status of infection (Xu et al., 1996). Interestingly, Tax is responsible for the constitutive activation of PI3K/Akt by impairing the association between the catalytic (p110) and the regulatory subunit (p85) leading to Akt Ser ^473^ phosphorylation in HTLV-1-infected cell lines (Peloponese and Jeang, 2006; **Figure 3**).

Pathways lying upstream of AP-1 are normally activated in response to external stimuli whereas Tax overrides this requirement (**Figure 3**). Additionally, the PI3K/Akt-AP-1 pathway had been involved in survival and is likely to be required for the immortalization of HTLV-1-infected cells (Jeong et al., 2005). Indeed, Tax even in the absence of NF-κB signaling is able to activate the Akt/PI3K pathway, which upon inhibition by dominant-negative mutants for Akt or for c-Jun abolishes the proliferation of Tax-transfected cell lines as well as the transformed phenotype (Peloponese and Jeang, 2006). AP-1 sites are Tax-inducible elements in different cellular genes, which promote cell proliferation and are associated with clinical characteristic features of ATL (**Figure 3**). Among target genes regulated by AP-1-binding sites, Tax activates growth-promoting cytokine genes, such as IL-2, IL-5, IL-13, as well as proinflammatory cytokines, such as IL-8 and TNF-a, and further induces the expression of immunosuppressors, like TGF-b1 and proenkephalin (PENK). This deregulation implies different combinations of dimerized AP-1 complex although they have not all been characterized as to their targeted promoters (Brady, 1992; Waldmann, 1996; Yamada et al., 1996; Jeang, 2001; Hall and Fujii, 2005).

Tax does not always activate cellular promoters bearing AP-1-binding site (for example, the collagenase gene) (Hall and Fujii, 2005). Rather, another mechanism, by which promoter could be regulated by Tax through AP1, is linked to the binding of Tax and c-Jun to an overlapping region corresponding to the KIX domain of CBP. This could induce a competition between these two proteins for CBP interaction and lead to the repression of c-Jun transcription activity by Tax (Van Orden et al., 1999; Van Orden and Nyborg, 2000). Although Tax induces the transactivation of the TGF-b1 promoter through AP-1 sites, Tax inhibits TGF-b1 signaling by reducing DNA-binding activity of Smad3 through a Smad3/c-Jun complex, which might be involved in the resistance of TGF-b1-induced growth inhibition observed in ATL cells, an important step in the pathogenesis of ATL (Arnulf et al., 2002). An opposite effect could be imputed to HBZ which could overcome the repressing effect of Tax on TGF-b1 signaling (Zhao et al., 2011). Tax might not solely account for the constitutive activity of AP-1, since a high activity of AP-1 has been detected in primary Tax^-^ leukemia cells of ATL patients (Fujii et al., 2000). These observations indicate that AP-1 is activated in HTLV-1-infected T cells through Tax-dependent and Tax-independent mechanisms.

### Hijacking of the AP-1 Signaling Pathway by HBZ

Within its antisense strand, HTLV-1 codes for a bZIP factor, which was appropriately named HBZ (HTLV-I bZIP factor) (Gaudray et al., 2002; Mesnard et al., 2006). HBZ can express under three isoforms: one unspliced form (usHBZ) and two alternatively spliced forms (HBZ-SP1 and HBZ-SP2) (Cavanagh et al., 2006; Murata et al., 2006) with HBZ-SP1 (or sHBZ) being the most abundant spliced variant. sHBZ is a 31-kDa protein with an N-terminal transcriptional activation domain, a central domain involved in nuclear localization, and a C-terminal bZIP domain (Gaudray et al., 2002; Mesnard et al., 2006). Sequence comparison between HBZ bZIP region and several bZIP factors clearly indicates that HBZ possesses a c-Fos-like bZIP domain although its DNA-binding domain lacks the consensus amino acid sequence bb-bN–AA-b(C/S)R-bb thought to be critical for DNA binding. HBZ interacts with all the members of the Jun family (JunB, c-Jun, and JunD), and differently regulates the transcriptional properties of the Jun family (Basbous et al., 2003; Hivin et al., 2005, 2007; Clerc et al., 2009).

### Sequestration of JunB and c-Jun in HBZ Nuclear Bodies Inhibits Their Transcriptional Activities

**FIGURE 4:**
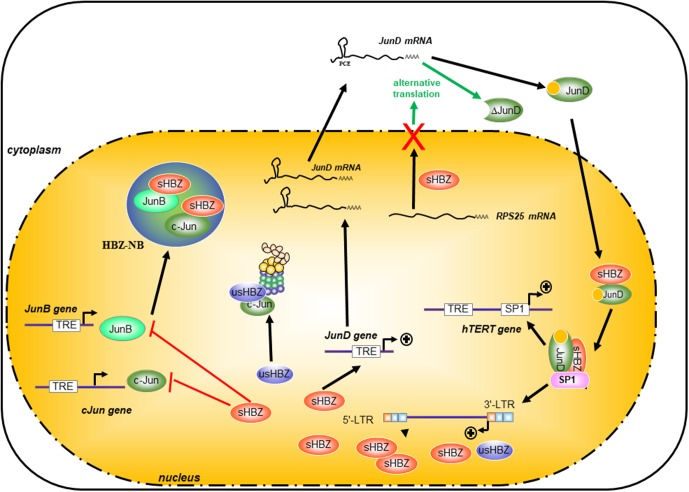
Differential effects of HBZ on the Jun family proteins. HBZ specifically interacted with all three members of the Jun Family (JunB, c-Jun, and JunD), but it regulates differently the transcriptional properties of the Jun family. HBZ dramatically suppressed c-Jun- and JunB-induced transcriptional activation from the AP-1 element by sequestering c-Jun and JunB into HBZ-NB and by decreasing the steady-state level of c-Jun and the stability of c-Jun protein in cells through a proteasome-dependent pathway. It is particularly interesting to note that HBZ has a different and opposite action on JunD expression. HBZ can stimulate the transcription of JunD and by nuclear retention of RPS25, HBZ allows the expression of an alternative isoform of JunD called ΔJunD. Furthermore, HBZ cooperates with JunD and sp1 to enhance transcription of the 3′-LTR and also the human telomerase reverse transcriptase gene (hTERT).

HTLV-1 basic leucine-zipper factor is a nuclear protein, which not only accumulates in specific nuclear bodies (called here HBZ-NBs) but is targeted to nucleoli (Hivin et al., 2005). Using a fluorescence recovery after photobleaching approach (FRAP) and an EGFP-tagged-HBZ, Hivin et al. (2007) have observed that the deletion of its leucine-zipper domain altered the rate of nuclear flux of HBZ, suggesting that HBZ heterodimerization partners are involved in controlling its own nuclear trafficking. Indeed, HBZ modifies the localization of JunB and targets JunB to the HBZ-NBs. Moreover, the relocalization of JunB into HBZ-NBs inhibits its transcriptional activity (Hivin et al., 2007; Clerc et al., 2009; **Figure 4**).

Although HBZ and c-Fos can both interact with c-Jun, they differ greatly in their abilities to activate transcription of AP-1-regulated genes. Indeed, the interaction of HBZ with c-Jun prevents this transcription factor from activating transcription of AP-1-dependent promoters by decreasing its DNA-binding activity (Clerc et al., 2009). The generation of different c-Fos/HBZ chimeras by region swapping indicates that the HBZ DNA-binding motif has an important impact on the transcriptional activity of both transcription factors in the presence of c-Jun (Hivin et al., 2007; Clerc et al., 2009). Indeed, the mutant HBZ-mutMD/DBD, for which specific residues present in the MD and DBD regions of HBZ were substituted for corresponding amino acids of c-Fos, showed a significant *in vitro* affinity for the AP-1-binding site TRE but remained unable to stimulate promoter activity of the AP-1-dependent collagenase gene *in vivo*. Like JunB, c-Jun is also relocalized to HBZ-NBs in the presence of HBZ-mutMD/DBD, while this transcription factor is diffusely distributed throughout the nucleus in the presence of HBZ-H14F (a construction in which the bZIP domain of HBZ-mutMD/DBD was replaced by the corresponding ZIP domain of c-Fos), suggesting that HBZ- inhibits c-Jun DNA-binding capacity *in vivo* mainly by its sequestration to the HBZ-NBs (Hivin et al., 2007; Clerc et al., 2009) (**Figure 4** blue circle).

### HBZ Promotes the Proteosomal Degradation of c-Jun

HTLV-1 basic leucine-zipper factor can also decrease the stability of c-Jun in cells and promote its degradation (Matsumoto et al., 2005). Indeed, in cells transfected with usHBZ, Matsumoto et al. (2005) observed that treatment with proteasome inhibitors but not with calpain inhibitors prevented the reduction in the steady state of c-Jun, suggesting that this HBZ-mediated reduction in c-Jun abundance could also occur through a proteasome-dependent pathway. It has also been suggested by Isono et al. (2008) that HBZ could act as a tethering factor between the 26S proteasome and c-Jun (**Figure 4**). However, c-Jun is less degraded by sHBZ (also called HBZ-SP1) than by usHBZ (Isono et al., 2008) and it remains unclear how both isoforms of HBZ could inhibit c-Jun through two different mechanisms.

### HBZ Activates the Transcriptional Activity of JunD

It is particularly interesting to note that HBZ has a different and opposite effects on c-Jun- and JunD-dependent transcription. Indeed, these two proteins belong to the same family of transcription factors, but they are very different proteins. In JunD expressing cells, HBZ is diffusely distributed throughout the nucleoplasm, while no HBZ-NBs are formed (Hivin et al., 2007). Interestingly, JunD is the only Jun family member, which can be activated by HBZ (Thebault et al., 2004; Kuhlmann et al., 2007). It is worth noting that the presence of the EQERRE motif in HBZ modulates JunD activity (Hivin et al., 2005). When the HBZ DNA-binding motif is substituted by the c-Fos modulatory domain, HBZ is no longer able to stimulate the transcriptional activity of JunD, although no alteration in the JunD DNA-binding activity is observed (Hivin et al., 2005). It is important to note that the abundance and activity of JunD increase in freshly isolated ATL cells concomitantly with an increase of HBZ expression (Nakayama et al., 2008; Terol et al., 2017). These observations suggest that HBZ modulates its own expression through a positive-feedback loop in resting cells that involves cooperation with JunD (**Figure 4**). Indeed, in HTLV-1-infected cells, HBZ enhances expression of JunD, which leads to the association of JunD and HBZ to Sp-1 bound to the 3′′-LTR-containing antisense promoter and, ultimately, to the activation of *hbz* transcription (Gazon et al., 2012).

In association with JunD and Sp-1, HBZ also activates the transcription of the human telomerase catalytic subunit gene (hTERT) (Kuhlmann et al., 2007). Telomerase, a ribonucleoprotein complex that extends telomeres which are essential for protecting chromosomal ends against end–end fusions or degradation (Cong et al., 2002; Brunori et al., 2005; Segal-Bendirdjian and Gilson, 2008). While mouse telomerase (mTERT) is activated in many normal tissues, human cells rarely spontaneously reactivate expression of the telomerase gene, as its expression is tightly regulated (Cong et al., 2002; Brunori et al., 2005; Segal-Bendirdjian and Gilson, 2008). However, 75–85% of cancer cells including ATL cells present an increase in telomerase expression and activity (Uchida et al., 1999; Brunori and Gilson, 2004; Brunori et al., 2005; Shay and Wright, 2011). Human telomerase is composed of a structural RNA component (hTERC), which contains an 11-base sequence complementary to the telomeric single-stranded overhang acting as a template for the synthesis of telomeric DNA. The other main component of the hTERT is its enzymatic reverse transcriptase subunit (Cong et al., 2002; Brunori et al., 2005; Segal-Bendirdjian and Gilson, 2008). Expression of *hTERT* is regulated mainly at the transcriptional level. The proximal 180 bp of the *hTERT* promoter, which does not contain any AP-1-binding site, is important for maintaining basal transcriptional activity and is thought to be the essential component for its regulation. Interestingly, Kuhlmann et al. (2007) have observed an increase in hTERT transcripts in cells co-expressing HBZ and JunD. Chromatin immunoprecipitation (ChIP) assays revealed that HBZ/JunD heterodimers interact with Sp1 and that activation of hTERT transcription by this trimer is mediated through Sp-1-binding sites present in the core region of the *hTERT* promoter (Kuhlmann et al., 2007).

We recently uncovered an additional mechanism used by HBZ to turn JunD from a growth suppressor to a tumor promoter (Terol et al., 2017). *JunD* is an intronless gene and produces two predominant isoforms by alternative initiation of translation, a 39-kDa protein (JunD-FL) through initiation from the first AUG codon and a shorter, 34-kDa JunD protein (ΔJunD) through the use of the second in-frame AUG codon (Hirai et al., 1989; Berger and Shaul, 1994; Short and Pfarr, 2002). Several studies indicated that JunD-FL and ΔJunD are differentially regulated through interactions with other nuclear proteins (Hirai et al., 1989; Berger and Shaul, 1994; Short and Pfarr, 2002). For example, menin, the product of the tumor-suppressor MEN-1 gene, represses JunD-FL transcriptional activity by interacting through its first 48 amino acids (Agarwal et al., 1999; Yazgan and Pfarr, 2001). Loss of menin expression or lost the ability of menin to bind JunD confers JunD with growth-promoting capabilities (Agarwal et al., 2003). ΔJunD does not bind menin, and its transcriptional activity is unaffected by menin overexpression (Yazgan and Pfarr, 2001). In addition, JNK binds and activates JunD-FL more efficiently than ΔJunD, even though both JunD isoforms contain a JNK-docking domain and three JNK phosphorylation target sites. It is interesting to note that freshly isolated ATL cells and HBZ-expressing T lymphocytes express both JunD isoforms (Yazgan and Pfarr, 2002).

JunD mRNA contains a third functional out-of-frame ORF (uORFs) positioned between the ATG of JunD-FL and ATG of ΔJunD (Short and Pfarr, 2002). Translation of downstream ORFs by uORF appears to be a common translational regulatory mechanism, as uORFs are present in two-third of mRNAs encoding oncoproteins and proteins that regulate important cellular processes. Alteration of protein expression levels by disruption or creation of uORF has been associated with the development of several human disease such as Alzheimer’s disease, acute myeloid leukemia, and breast cancer (Short and Pfarr, 2002; Zhou and Song, 2006; Wethmar et al., 2010; Barbosa et al., 2013). HBZ relieves uORF translational control by reducing the cellular abundance of RPS25, a ribosomal protein known to play a key role in several alternative translation mechanisms (Nishiyama et al., 2007). Using an immortalized fibroblast cell line model, we found that ΔJunD exhibits growth-promoting and -transforming activities that are enhanced in presence of HBZ (Terol et al., 2017). In summary, it has been proposed that HBZ/JunD heterodimers induce down-regulation of lymphocyte activation and viral transcription to favor viral latency and persistence of the infected cells. Future studies will aim to clarify how HBZ/JunD and/or HBZ/ΔJunD coordinately drive cell fate toward cellular transformation.

## Perspectives and Conclusion

Activating protein-1 family members have both overlapping and unique roles, and the transcriptional activity of the AP-1 dimer functions in a tissue-specific fashion (Shaulian, 2010). With respect to this important fact, recent studies have included the analysis of expression and/or activity of all Jun and Fos family members. Thus, it has been demonstrated that malignant transformation and tumor progression is accompanied by a cell-type-specific shift in AP-1 dimer composition (Verma et al., 1990; Radler-Pohl et al., 1993; Karin et al., 1997; Tulchinsky, 2000; Shaulian and Karin, 2002; Eferl and Wagner, 2003; Milde-Langosch, 2005; Hernandez et al., 2008; Shaulian, 2010). Those studies support a model, in which a shift in the expression pattern of the Fos family members is a crucial step in carcinogenesis and/or tumor progression (Tulchinsky, 2000; Milde-Langosch, 2005). Indeed, while uninfected CD4+ T-lymphocytes express mainly c-Jun and c-Fos proteins, ATL-leukemic cells are expressing JunD and Fra-2 (Nakayama et al., 2008; Terol et al., 2017). Due to their lack of a trans-activating domain, it has been suggested that Fra-1 and Fra-2 might exert anti-tumor effect and inhibit tumor cell growth. Yet, recent studies point to a positive effect of Fra-1, and partly Fra-2, on tumor growth (Young and Colburn, 2006; Milde-Langosch et al., 2008; Nakayama et al., 2008; Davies et al., 2011; Higuchi et al., 2013; Wang et al., 2014; Gupta et al., 2015).

Indeed, gene substitution experiments in mice have shown that growth retardation and osteoporosis observed in c-Fos null-mice were rescued by Fra-1 overexpression, although Fra-1 failed to induce expression of c-Fos target genes, such as MMP13 and vimentin (Young and Colburn, 2006). This observation is in line with results obtained in various cancer cell types, in which Fra-1 alters the biological behavior of the cells without directly activating AP-1-responsive promoters (Zerbini et al., 2003; Young and Colburn, 2006). Surprisingly, in most of the clinical tumor samples analyzed so far, Fra-1 expression is lower than in normal cells, and the protein is poorly phosphorylated (Zerbini et al., 2003; Young and Colburn, 2006). These observations raised several interesting questions on the true role of Fra-1 in oncogenesis and tumor progression. Indeed, whether the low level Fra-1 expression is due to tumor heterogeneity and if expression Fra-1 in specific clones within the tumors have a similar effect to what is seen in experimental systems and contributes to local invasion and metastasis should be further studied. In contrast to the bulk of data available on the function of c-Fos and Fra-1 in carcinogenesis, far less is known on the role of other Fos family members (FosB, FosB2, deltaFosB2, and Fra-2), which are often found expressed in high levels in cancer tissues. Further study of the role of all Fos proteins in carcinogenesis will be of great importance.

## Author Contributions

J-MP and HG wrote the first draft of the manuscript. HG, BB, J-MM, and J-MP wrote sections of the manuscript. All authors contributed to manuscript revision, read, and approved the submitted version.

## Conflict of Interest Statement

The authors declare that the research was conducted in the absence of any commercial or financial relationships that could be construed as a potential conflict of interest.
